# What Does the Human Olfactory System Do, and How Does It Do It?

**DOI:** 10.1146/annurev-psych-042023-101155

**Published:** 2023-10-03

**Authors:** Gülce Nazlı Dikeçligil, Jay A. Gottfried

**Affiliations:** 1 Department of Neurology, University of Pennsylvania, Philadelphia, Pennsylvania, USA;; 2 Department of Psychology, University of Pennsylvania, Philadelphia, Pennsylvania, USA

**Keywords:** human olfaction, odor perception, fMRI, functional magnetic resonance imaging, intracranial electroencephalogram, sensory neuroscience, human peripheral olfactory system

## Abstract

Historically, the human sense of smell has been regarded as the odd stepchild of the senses, especially compared to the sensory bravado of seeing, touching, and hearing. The idea that the human olfaction has little to contribute to our experience of the world is commonplace, though with the emergence of COVID-19 there has rather been a sea change in this understanding. An ever increasing body of work has convincingly highlighted the keen capabilities of the human nose and the sophistication of the human olfactory system. Here, we provide a concise overview of the neuroscience of human olfaction spanning the last 10–15 years, with focus on the peripheral and central mechanisms that underlie how odor information is processed, packaged, parceled, predicted, and perturbed to serve odor-guided behaviors. We conclude by offering some guideposts for harnessing the next decade of olfactory research in all its shapes and forms.

## INTRODUCTION

The purpose of the olfactory system is to extract behaviorally meaningful information from volatile molecules in the environment. Odors emanating from sources near and far can provide organisms with predictive cues about the presence of predators, availability of mates, identification of kin, and proximity of food. That said, the functions of the olfactory system are not limited to mere survival and reproduction per se but extend to all manner of behavior involving odorous compounds, such as odor-guided navigation, associative learning, and long-term memory. To inform and support such a diverse range of behaviors, the olfactory system has evolved to detect, identify, and differentiate a great many volatile molecules. Of course, these olfactory functions operate in concert with the other senses to provide a behaviorally salient summary of the environment to optimally guide behavior. To this end, the relative contribution of olfactory information for guiding behavior vis à vis sensory information from other modalities depends very much on the ecological niche of the species.

For humans, smells often take a back seat to the boisterous immediacy of sight, sound, and touch—sensations that are ever present and dominating in the conscious perceptual experience and are frequently relied upon for day-to-day functions. Yet, this experientially muted nature of human olfaction cannot be attributed to its blunted abilities or lack of importance for human survival and well-being (Croy et al. 2014, Laska 2017, Laska et al. 1999, McGann 2017, Stevenson 2010). Indeed, the human nose can detect a fantastic number of volatiles, with estimates ranging from ten thousand to one trillion molecules (Bushdid et al. 2014), though the head-spinning estimate of one trillion has received considerable pushback (Gerkin & Castro 2015, Meister 2015), and the exact number of distinct odorous molecules detectable and differentiable by the human nose remains unknown. Additionally, humans can detect some compounds at concentrations as low as 10 parts per billion or less (Cain et al. 2007, Sarrafchi et al. 2013) and, furthermore, can even discriminate between two odor molecules that are mirror images of one another (Laska & Teubner 1999), highlighting the sensitivity and specificity of the human olfactory system.

Beyond the sensory capabilities of the human nose, it is fair to state that the significance of olfaction for humans is best appreciated in its absence. Temporary or permanent loss of smell (i.e., anosmia), a condition intimately familiar to millions of people around the world due to the COVID-19 pandemic (Parma et al. 2020), introduces significant complications when it comes to instrumental and personal activities of daily life. The inability to enjoy the flavors of a meal, to perceive that the milk might be rancid, or to assess if one’s body odor is perennially offensive creates significant stress and is associated with a higher risk of depression (Croy et al. 2014, Neuland et al. 2011, Nordin et al. 2011). At the same time, while scents are seldom the sole focus of human attention, they discreetly but frequently color our perceptual experience (Croy et al. 2016; Zhou et al. 2010, 2018). It is enough to imagine how impoverished our daily experience becomes in the absence of scents. For example, how does it feel to stroll in a forest without detecting the smell of geosmin that creates the familiar earthy scent after a rainstorm? Or to sit down at the dinner table without being cocooned by the comforting odors of roasted pork, a glass of white burgundy, rhubarb crisp, and the ensemble of ordinary scents that uniquely defines home? Such examples illustrate, from a phenomenological perspective, how sterile the human experience becomes in the absence of olfaction, the muted sense that discreetly shapes our perceptions, emotions, and memories.

Humans inhabit odor-rich landscapes awash with diverse odor sources, each emitting tens to hundreds of different odorous molecules. This olfactory cacophony is more the rule than the exception, as very few scents in the natural world are derived from just one single molecule. For example, the aroma of pressure-cooked pork liver is comprised of 179 molecules (Mussinan & Walradt 1974); roughly 600 compounds create the full rich smell of chocolate (Aprotosoaie et al. 2016, Ziegleder 2017); and ~50 unique odor volatiles contribute to the signature scent of popcorn smell, which, ironically and fascinatingly, has been studied by Dr. Ron G. Buttery at the United States Department of Agriculture (Buttery et al. 1997). The olfactory system de facto knits all of these disparate compounds into integrated wholes to create odor percepts, and much like the individual brush strokes that disappear in favor of the complete scene in an impressionist painting, the individual odor molecules are also interpreted in the context of the holistic ensemble of odorous molecules (i.e., odor objects). We contend that these odor objects are every bit as much sensory objects as their visual counterparts, although the notion of sensory objects composed of volatile components creates a surprising amount of confusion among vision scientists.

A unique feature of odorants, in contrast to sensory stimuli in other modalities, is the extent to which they can transcend both space and time. For example, a dog may leave a telltale aromatic calling card on a grassy field, and the emanating volatile cues can be carried via air currents to a distal receiver. Unlike the evanescent properties of a visual scene that may come and go, that same olfactory calling card may linger beneath an oak tree for several days, creating a temporal catchment that can host odor information for newly arriving visitors. This ability of odor cues to transmit information across time and space also introduces a large degree of variance into the signal: Volatile components evaporate, scents degrade, and the persistent presence of nearby odorants turns into intermittent bouts when transmitted via air currents across long distances. Despite this large degree of noise, the human olfactory system can do a fairly satisfying job of narrowing down what the nose is actually smelling. The faint scent of roasted coffee lingering in the streets of the Grand Bazaar in Istanbul can still be recognized readily amid a dizzying olfactory backdrop of spices, leather goods, and other delicacies. How the olfactory system can detect, identify, and characterize odor objects with high fidelity amid such a large degree of ambiguity remains mostly unknown.

The overarching goal of this review is to provide an up-to-date account of the neuroscience of human olfaction literature, principally emphasizing work over the last 10 to 15 years, focusing on odor processing in the healthy brain, and featuring emerging new areas and themes of research. To begin, we focus on the question of olfactory receptors (ORs) and the ligands that bind them (i.e., odorous molecules). Subsequent sections emphasize both spatial and temporal processing of odor information based on complementary approaches utilizing behavioral paradigms, task-based functional magnetic resonance imaging (fMRI), and human electrophysiological recordings. We would like to note that due to space limitations, we are unable to feature many other exciting lines of research, such as the role of odor cues in human social communication, the relationship between olfaction and language, and the anatomical and functional changes that occur in the olfactory system in response to viral infections and neurodegenerative disorders.

## BRIEF PRIMER OF HUMAN OLFACTORY ANATOMY

**Figure 1:**
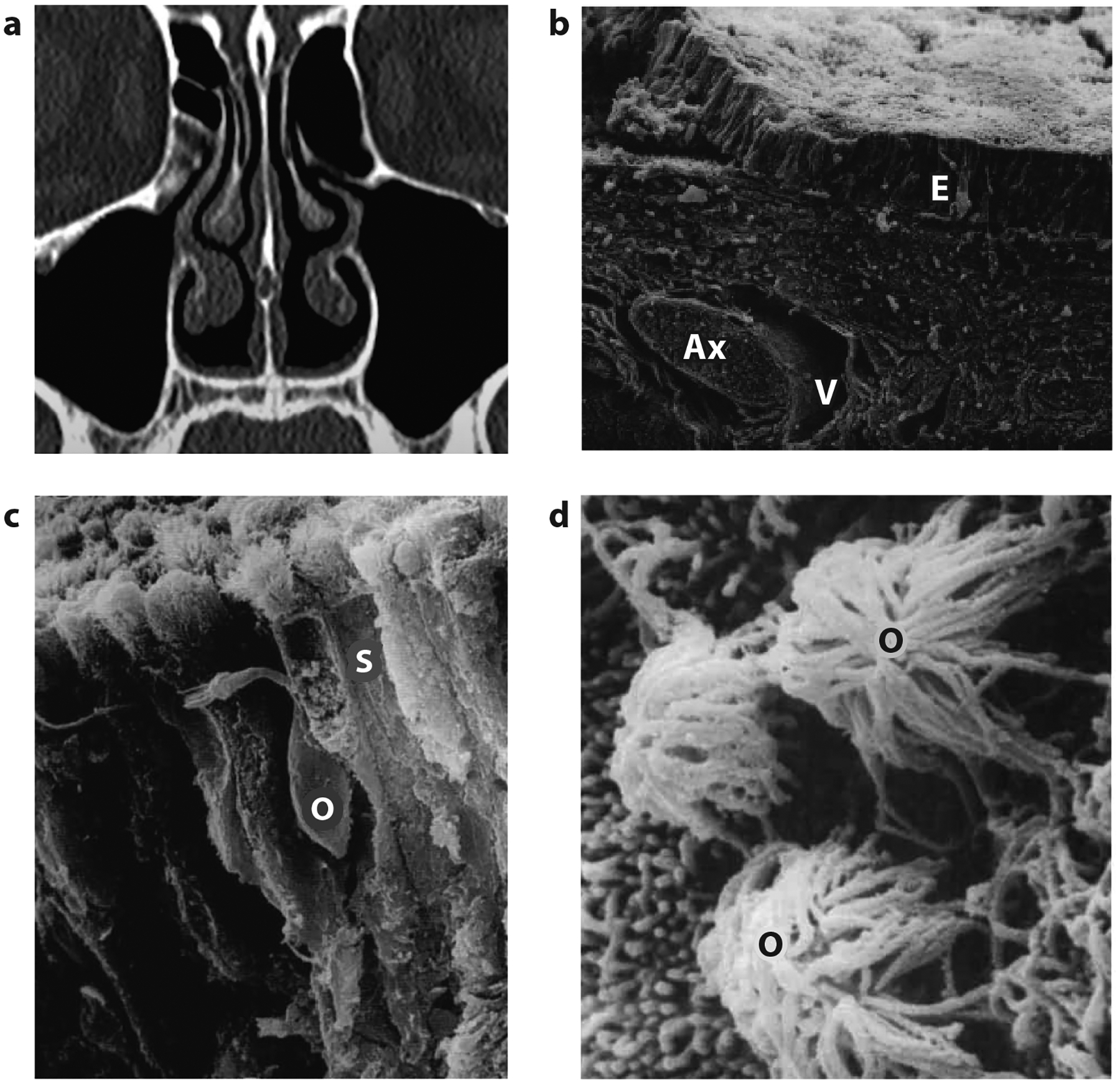
(*a*) Coronal cross section of the human nose illustrates the nasal turbinates and the surrounding nasal cavity. Olfactory epithelia are mainly distributed across the dorsal portions of the nasal cavity. (*b*) Low-power three-dimensional scanning view of the olfactory epithelium and lamina propria. The olfactory epithelium (labeled E) overlies a thick connective tissue lamina propria that contains olfactory axon fascicles (Ax) and blood vessels (V) (×248 magnification). (*c*) Supporting cells (labeled S) are columnar cells and extend the full depth of the epithelium. An olfactory sensory neuron (O) with its dendrite can be seen among supporting cells (×1,241 magnification). (*d*) High-power scanning view of the epithelial surface at the transition zone between olfactory and nonsensory epithelia. A cluster of olfactory sensory neurons (labeled O) can be seen among the short and long microvilli surface of nonsensory cells (×10,000 magnification). Panels *b* and *c* adapted with permission from Morrison & Costanzo (1990); panel *d* adapted with permission from Morrison & Costanzo (1992).

**Figure 2:**
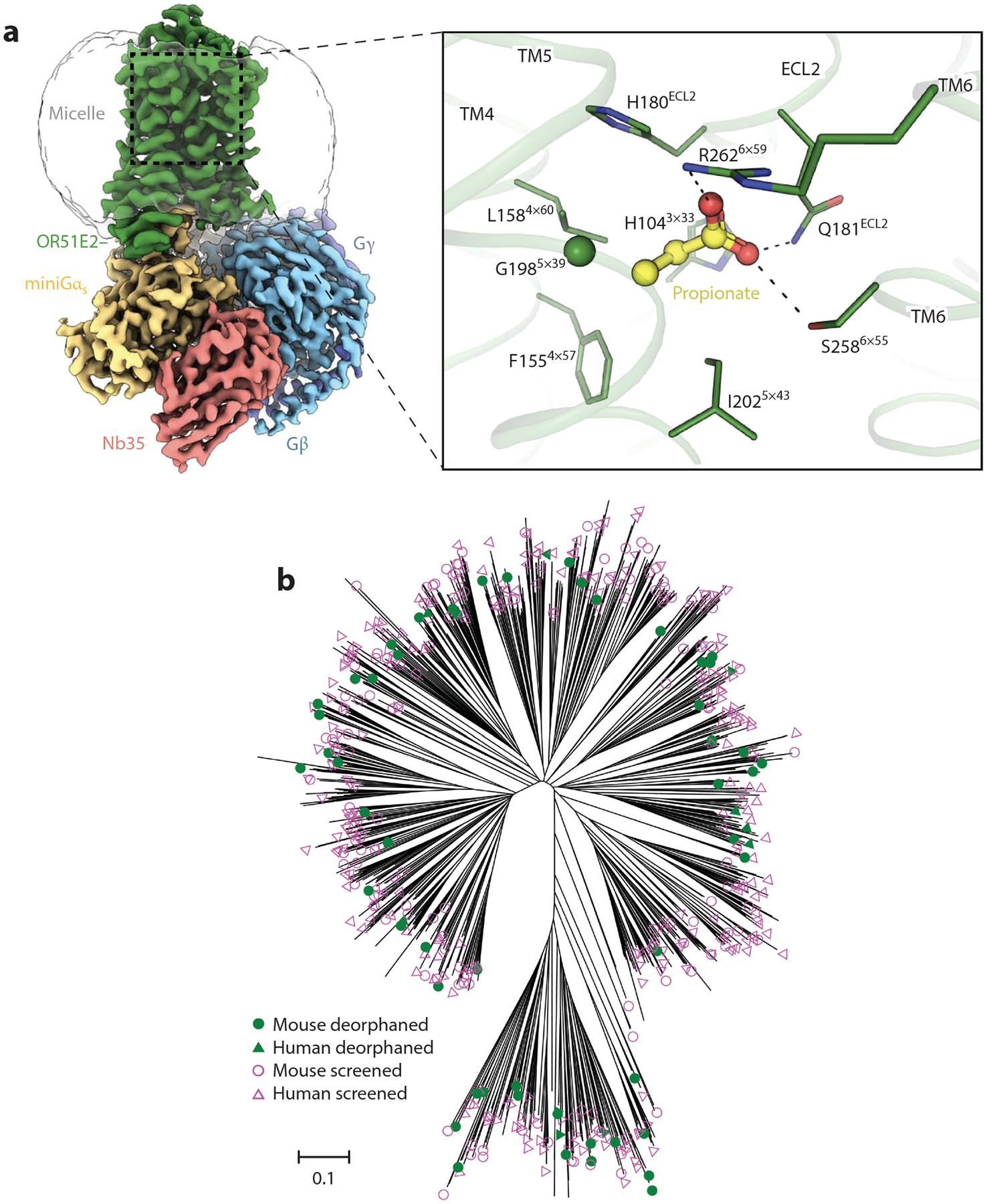
(*a*) Structure of an odor molecule bound to a human odorant receptor called OR51E2. This receptor is specific for sensing short-chain fatty acids such as acetate and propionate. On the left is a three-dimensional map obtained by cryo-electron microscopy of OR51E2 bound to the intracellular signaling proteins Gβ, Gγ, and a miniaturized version of Gα_s_ together with a nanobody (Nb35) and detergent micelle to stabilize the complex. On the right is a magnified image of the propionate odorant (*yellow* and *red* structure) buried in OR51E2 to activate the receptor. Propionate interacts with various amino-acid residues (labeled) in the odorant-binding pocket of OR51E2 (*dashed lines* indicate ionic and hydrogen bonds). Panel adapted with permission from Billesbølle et al. (2023). (*b*) A phylogenetic tree of all 464 receptors in the screening library as well as 1,425 intact mouse and human olfactory receptors. Receptors used in the mixture-screening phase are labeled with open magenta symbols; receptors found to have at least one agonist in this study are labeled with closed green symbols. Mouse receptors are labeled with circles; human receptors are labeled with triangles. Unlabeled lines represent untested receptors. Panel adapted with permission from Saito et al. (2009). Abbreviation: TM, transmembrane.

The molecular cascade of events that ultimately materializes into an odor percept begins at the nose, where inhaled air disperses odor molecules across the nasal turbinates and ensures that odorants maximally engage the olfactory sensory neurons (OSNs) positioned at the dorsal extent of the nasal cavity (Figure 1). Odor molecules bind to the ORs on the ciliated dendrites of OSNs, leading to conformational changes in the receptors (Figure 2*a*) and initiating a cellular response. Axons of the OSNs cross through tiny perforations in the cribriform plate to the central nervous system and synapse onto the glomeruli in the ipsilateral olfactory bulb (OB), where incoming axons from the olfactory epithelium (OE) on the presynaptic side of the circuit meet with mitral and tufted cells on the postsynaptic side of the circuit. This cellular organization is highly stereotyped in rodents, such that all of the olfactory axons expressing the same OR converge onto ~2 glomeruli, likely in order to optimize signal amplification and gain control. The extent to which the human OB is organized in a similar fashion is currently not known.

**Figure 3:**
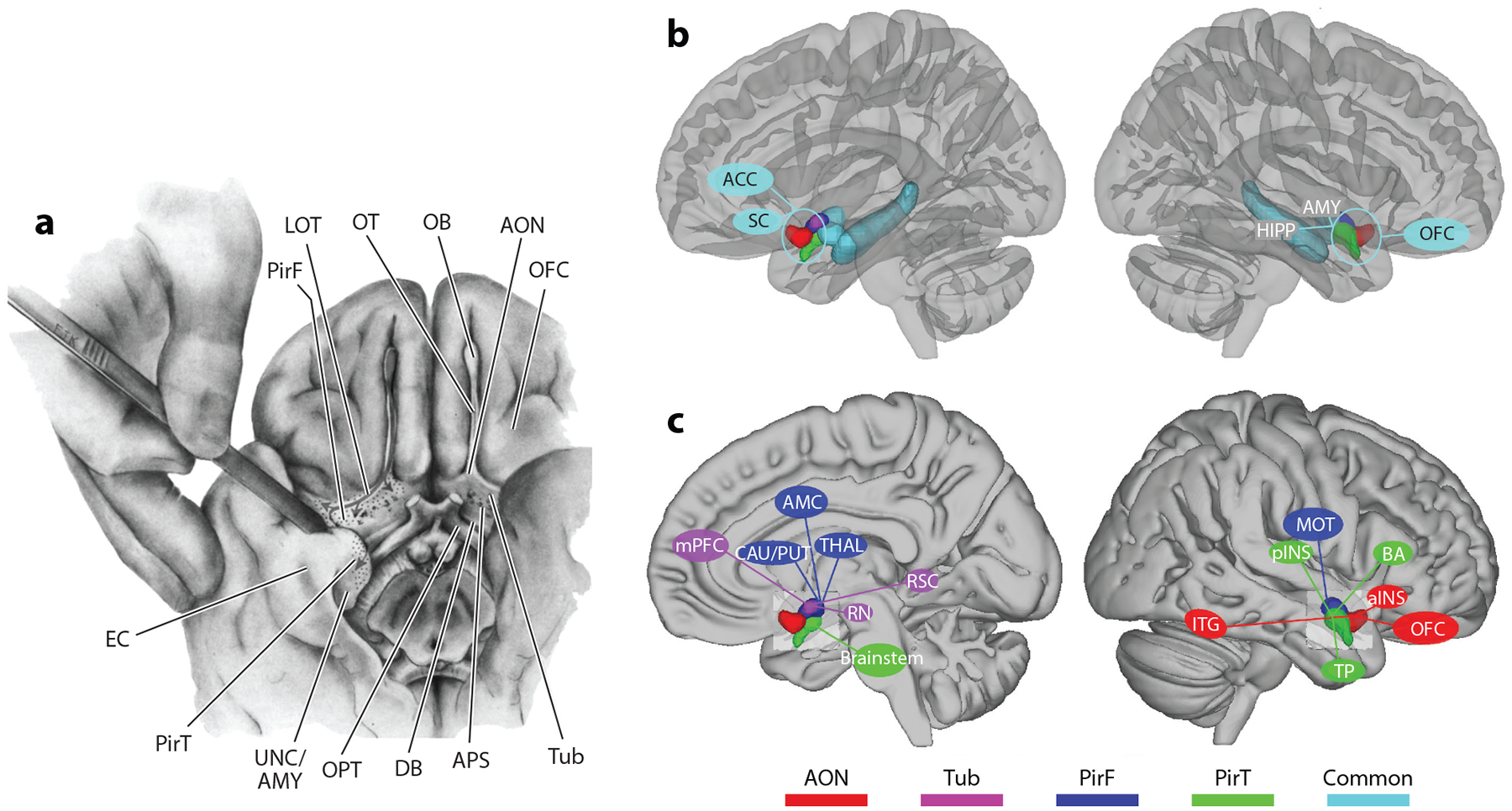
(*a*) Anatomical illustration of the human basal forebrain and medial temporal lobes, depicting the olfactory tract, its principal projections, and surrounding nonolfactory structures. Panel adapted with permission from Heimer (1983, figure 159); copyright 1983 Springer. (*b*) Schematic summary of the functional connectivity of human primary olfactory cortex subregions. The figure illustrates the brain regions that are commonly connected to all subregions of the olfactory cortex. Panel adapted with permission from Zhou et al. (2019a). (*c*) Same as panel *b* except that the schematic illustrates the brain regions that are uniquely connected to each subregion of olfactory cortex. Panel adapted with permission from Zhou et al. (2019a). Abbreviations: ACC, anterior cingulate cortex; aINS, anterior insular cortex; AMC, anterior mid-cingulate cortex; AMY, amygdala; AON, anterior olfactory nucleus; APS, anterior perforated substance; BA, Broca’s area; CAU, caudate; DB, diagonal band; EC, entorhinal cortex; HIPP, hippocampus; ITG, inferior temporal gyrus; LOT, lateral olfactory tract; MOT, motor area; mPFC, medial prefrontal cortex; OB, olfactory bulb; OFC, orbitofrontal cortex; OPT, optic tract; OT, olfactory tract; pINS, posterior insular cortex; PirF, frontal piriform cortex, PirT, temporal piriform cortex; PUT, putamen; RN, red nucleus; RSC, retrosplenial cortex; SC, subcallosal cortex; THAL, thalamus; TP, temporal pole; Tub, olfactory tubercle; UNC/AMY, uncus with amygdala situated beneath.

Downstream from the OB, two sets of projection neurons, the mitral and tufted cells, coalesce into the olfactory tract, one on each side of the brain, and project ipsilaterally to a wide number of brain areas, including the anterior olfactory nucleus (AON), olfactory tubercle, piriform cortex, amygdala, and entorhinal cortex (Figure 3*a*). Collectively, the brain areas receiving direct input from the OB compose the primary olfactory cortex. Piriform cortex receives the densest projections from the OB and is the most extensively studied subregion of the primary olfactory cortex. The connectivity of the olfactory system differs from other sensory modalities in two important ways:(*a*)Odor information reaches olfactory cortex within just two synapses, without an obligatory thalamic relay; and (*b*) odor information is processed in parallel streams downstream of the OB.

Histologically, the AON, a collection of cell clusters scattered along the caudal olfactory tract and orbitofrontal cortex (OFC), is fairly well understood in rodents but is challenging to identify in humans. For these reasons, it remains an open question whether the AON plays a relevant functional role within the human olfactory system. At the point where the lateral part of the olfactory tract wraps onto the medial temporal surface (uncus), major projections disseminate to piriform cortex, amygdala, and rostral entorhinal cortex, all of which are substantially interconnected via associational intracortical fiber systems. The OT forms a prominent bump in rodents, though it, too, is difficult to visualize in humans. Curiously, recent diffusion tensor imaging research tentatively suggests that this structure is situated along the posteriormost segment of the medial orbital cortex within the anterior perforated substance, though confirmatory understanding at the cellular or molecular level is lacking (Echevarria-Cooper et al. 2022). Lastly, resting-state functional imaging of the olfactory system has suggested that subregions of primary olfactory cortex are functionally connected to OFC, insula, amygdala, thalamus, basal ganglia, and hippocampus, among others (Zhou et al. 2019a) (Figure 3*b*). Interestingly, this work revealed that subregions of the olfactory cortex also display unique functional connectivity (Figure 3*c*), likely reflecting the distinct roles these structures may play in odor perception and odor-guided behaviors. It is important to note that bona fide synaptic connections within the human olfactory system remain poorly understood, with the bulk of knowledge inferred from rodents (Lane et al. 2020).

## WHAT DOES THE MOLECULE TELL THE NOSE?

### Olfactory Receptor Basics

Olfactory processing begins with the binding of an odorous molecule to an OR in the nose. This simple dyad between odorant and receptor plays out in all mammals, though marked qualitative and quantitative differences in the types and diversity of receptors endow each species, including *Homo sapiens*, with a unique repertoire of olfactory abilities (Saraiva et al. 2019). However, this crucial first step of olfaction was not well understood until seminal work by Linda Buck and Richard Axel identified the large multigene family encoding a diverse array of G-protein coupled receptors specialized for odor detection in rodents (Buck & Axel 1991). Buck and Axel’s findings suggested that the olfactory system utilizes this large variety of ORs in the OE to detect and discriminate different odor molecules (Figure 2*b*).

Buck and Axel’s discovery galvanized the field and inspired a plethora of groundbreaking studies focused on the molecular and genetic principles of ORs in both rodents and humans. By the early 2000s the basic tenets of the peripheral olfactory system were well in place (Buck 2004), establishing that (*a*) ORs reside mainly in the OE; (*b*) each OSN expresses only one type of OR; (*c*) each odorant can bind to multiple ORs, and each OR in turn can also bind multiple odor molecules, albeit with differing affinities, and as a result, odor stimuli activate distinct subset of ORs, creating a combinatorial olfactory code; (*d*) the human genome has nearly 900 distinct OR genes but only about 400 of them are functionally intact and odor responsive; and (*e*) there is great variation within humans in their olfactory sensitivity, potentially related to specific single nucleotide polymorphisms (SNPs) in OR genes. However, it must be stated that the extent to which the organizational principles of the rodent and human olfactory system resemble one another on a finer-grained level is still not well understood (Lane et al. 2020).

Methodological advances by Saito and colleagues enabled more robust and stable expression of ORs on the cell surface (Saito et al. 2004) and facilitated more efficient deorphanization of ORs (i.e., identification of odor molecules that bind to a given OR). In a subsequent study leveraging this new approach (Saito et al. 2009), Saito and coauthors tested a large library of both human and mouse ORs against a panel of 93 odorants and found that human ORs were more sensitive to their agonists compared to the mouse ORs, although the extent to which this finding can be generalized to the rest of the human and mouse ORs is currently unknown.

### How Variability in Odorant Receptor Genetics Influences Odor Percepts

An exciting line of studies in the past 15 years has aimed to understand how variations in OR genes contribute to perceptual variability across individuals. Addressing this question is challenging due to the multiplexing of receptor–ligand interactions: Each OR can be activated by many ligands, and each ligand can bind to more than one OR, thus providing the olfactory system with a complex combination of options for detecting odorants. Keller and colleagues took advantage of a very narrowly tuned human odorant receptor, OR7D4, which was selectively activated in vitro by androstenone and a related odorous steroid, androstadienone (both musk-like in odor quality), but did not respond to a panel of 64 other odorants (Keller et al. 2007). Notably, subjects with SNPs impairing OR7D4 function were less sensitive to androstenone and androstadienone and perceived the odors to be less unpleasant compared to subjects lacking these polymorphisms. Subsequent studies focusing on different ORs provided additional evidence that genetic variations in ORs could alter the perception of their ligands; loss of function in OR11H7P, OR2J3, and OR5A1 receptors was associated with increased detection thresholds for their respective ligands isovaleric acid (Menashe et al. 2007), cis-3-hexen-1-ol (McRae et al. 2012), and beta-ionone (Jaeger et al. 2013).

Using a larger set of human ORs, Mainland et al. (2014) also investigated how genetic variations are associated with perceptual variations. Leveraging the high throughput approach developed by Saito et al. (2004), the authors identified agonists for 18 new ORs that were previously orphaned. Notably, 63% of these ORs had SNPs that altered their function in in vitro assays, raising the possibility that these polymorphisms could influence OR function in vivo and, subsequently, odor perception across individuals. The authors found that variations in the OR10G4 genotype explained 15% of the observed variation in perceived odor intensity and over 10% of the variation in perceived valence for the high-affinity ligand guaiacol (smoky smell). Interestingly, when the authors tested lower affinity agonists for this receptor (i.e., vanillin and ethyl vanillin), they found that perceptual variation across individuals was not associated with the variations in the OR10G4 gene. Subsequent large-scale work by Trimmer et al. (2019) has reported congruent findings with seven new OR–odorant pairs in which genetic variation in the ORs was associated with changes in odor intensity and pleasantness perception.

Collectively, these studies demonstrate a direct link between single ORs and perception of monomolecular odorants; however, it is important to note that such strong causal relationships between genetic variations and odor percepts are more likely to be the exception rather than the rule. Most scents encountered in daily life are composed of dozens of monomolecular odorants, and each molecule typically binds to more than one OR. Furthermore, each OR can be bound to many ligand types. This combinatorial code provides redundancy and can endow the olfactory system with robustness in the face of SNPs, enabling detection and recognition of odor objects despite genetic variations in a small number of receptors. Additionally, while genetic variation is certainly one factor that can contribute to perceptual variability, many other behavioral and cognitive factors, ranging from culture and language to attention and satiety, can influence odor perception both within and across individuals.

### What Does Odorant Structure Have to Do with the Odor Percept?

Considering the vast number of odorous molecules, assembling a systematic understanding of how physical properties of odors map onto odor percepts remains no easy feat (Rossiter 1996). Furthermore, the puzzling challenges that humans confront when describing their subjective odor experience introduces an additional layer of difficulty to establishing robust links between the stimulus and the percept. To bring a comprehensive formalism to the olfactory perceptual field, Andrew Dravnieks pioneered the *Atlas of Odor Character Profiles* in 1985 (Dravnieks 1985). The atlas provides odor quality evaluations obtained from 140 participants who rated 159 odorants based on 146 different semantic descriptors such as “sooty,” “burnt rubber,” and “wet dog.” For each odorant and descriptor, subjects provided a rating ranging between 0 and 5, where 5 represents a perfect match between the odor and descriptor. The averaged ratings were shown to provide stable multidimensional odor profiles that were similar across different sets of raters (Dravnieks 1982). This scale offers a robust approach to characterizing and quantifying the subjective odor experience and remains widely in use.

Studies in recent years have compiled and leveraged large data sets containing hundreds of molecular and perceptual features to determine if a systematic relationship exists between odor structure and odor percept (Keller et al. 2017, Khan et al. 2007, Koulakov et al. 2011, Ravia et al. 2020, Snitz et al. 2013). Khan and colleagues used the odor ratings provided in the *Atlas of Odor Character Profiles* (Dravnieks 1985) to create a perceptual odor space. Using dimensionality reduction approaches and linear models, the authors found that odor pleasantness explained 30% of the variance in this perceptual space (Khan et al. 2007). Interestingly, the physicochemical features that accounted for the largest variance in the molecular space were correlated with odor pleasantness, leading the authors to reason that physicochemical properties may be an important predictor of odor pleasantness. However, it is important to note that molecular properties could not account for the other 70% of variance in odor perception. A capstone to this line of research materialized in 2017, taking the form of a crowdsourced DREAM Olfaction Prediction Challenge where machine-learning methods were used to predict sensory attributes of molecules based on their chemoinformatic features (Keller et al. 2017). This call to arms (to noses, actually) involved 22 different teams across multiple countries, who were invited to solve the stimulus-percept problem. The authors observed that physicochemical properties could be used to predict the pleasantness of novel molecules using the algorithms trained on the existing pairings. The best models were based on random-forest and regularized linear models; model predictions for intensity and pleasantness were also highly correlated. On the other hand, it was more difficult to predict the 19 semantic predictors, with correlation coefficients of 0.21 at the individual level and 0.41 at the population level.

It is possible that the observed links arise because odorants from similar odor sources (e.g., flowers) have correlated molecular features (Dudareva et al. 2004, 2006; Tholl et al. 2011), and the perceptual qualities they share arise from their shared ecological function (i.e., flowers eliciting approach behavior) rather than from similarities in their molecular features per se. In other words, the olfactory system might be grouping odor objects based on the functional and ecological relevance of the odor source rather than the molecular features of odorants, but the underlying correlations in the molecular features of odors coming from similar sources might lead to predictable patterns between molecular features and perceptual qualities of odorants.

## DISTRIBUTED PATTERNS OF ODOR ACTIVITY IN THE HUMAN BRAIN

In moving aside from odor molecules and the receptors that bind them, this section of our review focuses on central mechanisms of olfactory processing in the human brain. As with any sensory system, the functions of the olfactory system extend beyond the mere detection of stimuli, as sensation is only a small step in the cascade of neural computations aimed at extracting meaning from odors in the context of an organism’s internal states and external milieu. For example, the unctuous aroma of a pizza pie may awaken a diner’s salivary glands ahead of a meal or can elicit disgust if recovering from food poisoning. There is also a great deal of perceptual trickery when it comes to how contextual cues influence olfaction (Bensafi et al. 2007, 2014; Djordjevic et al. 2008; Herz 2003; Morrot et al. 2001): Place one odorous substance into a paper bag and label it “parmigiano reggiano,” and there will be quick consensus that this is indeed a fine cheese aroma, but relabel the paper bag as vomit, and that very same odorant will suddenly become unbearable. Such examples illustrate the importance of external context, multimodal cues (Manesse et al. 2020, Seo & Hummel 2011), and internal state (Shanahan & Kahnt 2022) for interpreting odor stimuli and highlight the integration of information across sensory, cognitive, mnemonic, and emotion-based networks. Here we feature some of the conceptual underpinnings that have steered our understanding of how odor information is processed, packaged, parceled, predicted, and perturbed in the human brain. Much of the research reviewed here would not have been possible without profound advances in MRI physics, novel statistical approaches, cutting-edge experimental paradigms, and those minor but essential ingenuities necessary for delivering odors reliably to the human nose while simultaneously measuring metrics of sniffing.

It is important to note that much of our understanding of odor processing in the human olfactory system derives from studies on piriform and orbitofrontal cortices. In contrast, studies on other primary olfactory regions (i.e., AON, olfactory tubercle, amygdala, and entorhinal cortex) have been relatively sparse, and neuroimaging studies of the human OB have been virtually impossible due to its small size and susceptibility to MRI signal dropout (see Fournel et al. 2020, Miao et al. 2021 for recent technical advances in human OB neuroimaging). Two independent case studies have reported the curious observation that subjects with seemingly absent OBs (based on MRI imaging) could nonetheless smell odor stimuli, and that the sniffing of odors elicited responses at the cortical level (Rombaux et al. 2007, Weiss et al. 2020). Insofar as the studies could not conclude whether the OBs were truly absent or simply existed in an altered form and location, their findings raise further questions about the structural variability of OBs and whether neuroimaging approaches can reliably capture human OB signals.

### Historical Background

In the lean pre-fMRI decades, there were few options for understanding how and where odor information was mapped onto the human brain and how these regions communicated with each other. The limited research principally relied on careful postmortem dissections (Allison 1954), lesion studies (Gordon & Sperry 1969, Potter & Butters 1980, Zatorre & Jones-Gotman 1991), or flash-in-the-pan electrophysiological recordings in the human OB (Hughes et al. 1969, 1970) and amygdala (Hughes & Andy 1979). The subsequent emergence of positron emission tomography (PET) scanning, followed by fMRI, kindled noninvasive studies of the healthy human brain and set the stage for the modern era of cognitive neuroscience.

It is important to state that the general framework of cognitive neuroscience research in the early 2000s was that of functional localization, where the overarching goal was to map perception, learning, decision making, and emotional states onto discrete punctate functional areas of the human brain, typically via the identification of voxels with the most statistically robust responses (i.e., univariate analyses methods). The field of human olfaction was no different in this regard, as the early questions centered on asking what areas of the brain encode smell information. Stepping into this brave new void, Zatorre et al. (1992) used PET techniques to characterize how the brain responds to scents. Subjects were placed into the PET scanner and were presented either with an odorless cotton wand or with a cotton wand saturated with one of eight different odorants. By comparing activation in the odor versus no-odor conditions, the authors identified significant and selective PET neural activity in piriform cortex and OFC. Fast-forward to 1997, and Sobel et al. (1997) developed a novel methodology for odorant delivery to subjects lying in the scanner and used this approach in their fMRI studies to disentangle regions activated by olfactory exploration (i.e., sniffing) versus regions activated by olfactory content (i.e., the perceived character or quality of the smell), revealing distinct regional differences in piriform cortex and OFC (Sobel et al. 1998). These groundbreaking studies were among the first to map odor-based percepts and behaviors onto specific regions of the human olfactory brain (see also Levy et al. 1997, Yang et al. 1997, Yousem et al. 1997).

### Odor Coding in Piriform Cortex

A central and rather thorny question, which remains as much a dilemma today as it was in the initial years of olfactory fMRI investigations, is the lack of a predictable relationship between odorant structure and odorant perceptual quality. Odorants sharing similar molecular structures may smell different from one another, while odors that are structurally distinct may smell similar. For example, molecules from five distinct structural classes all evoke a musky percept, but the enantiomers of the said molecules do not evoke this musky odor quality (Sato-Akuhara et al. 2023). The extent to which neural activity in human piriform cortex reflects odor quality perception versus odorant structure per se is largely open to debate. To address this question, we implemented an olfactory version of a cross-adaptation paradigm (inspired by visual cross-adaptation studies; Buckner et al. 1998, Kourtzi & Kanwisher 2001, Winston et al. 2004) in an effort to dissociate representations of odorant structure (the molecule) from the olfactory percept (the smell). To this end, human subjects were instructed on each trial to make two successive sniffs to pairs of odorants that varied either in perceptual quality (lemon-like or vegetable-like) or in the molecular functional group (alcohol or aldehyde) (Gottfried et al. 2006). The central prediction was that if piriform cortex represents odor quality independent of functional group, then sequential presentation of qualitatively similar odorant pairs should elicit decreased blood-oxygen-level-dependent (BOLD) activity compared to qualitatively dissimilar pairs. These results highlighted a double dissociation in piriform cortex, whereby posterior regions encoded quality (but not structure) and anterior regions encoded structure (but not quality), implying that the perception of an odor is a synthetic process by which dozens to hundreds of volatile organic compounds generate the full expression of an odorous object.

**Figure 4:**
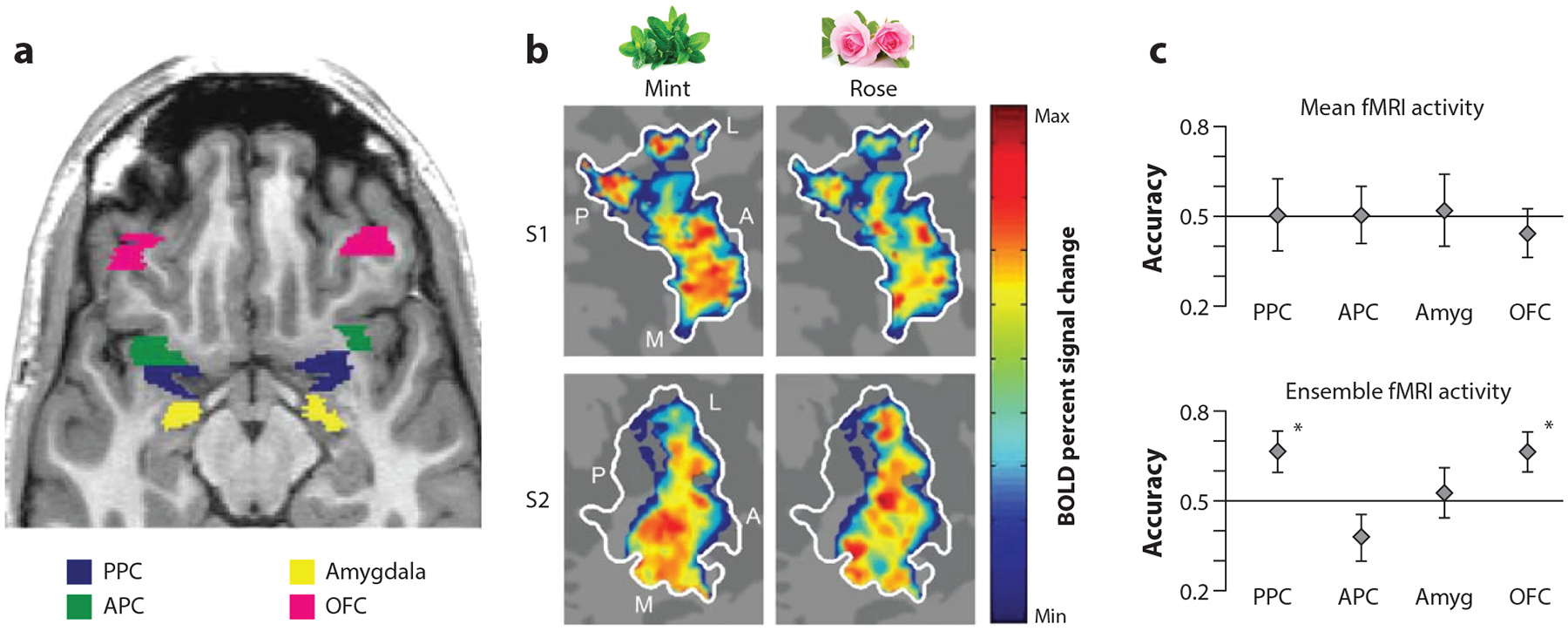
(*a*) Axial slice of a T1-weighted structural scan showing anatomically defined regions of interest. Subsets of voxels from these brain regions were used in the pattern analyses. (*b*) Odorant-specific spatial maps in left PPC. The three-dimensional representations of odor-evoked activity in PPC from two subjects were projected onto two-dimensional (flat) maps to visualize voxel-wise odor patterns on a single plane. Maps depict the odorant-evoked BOLD percent signal change in all odor-active voxels (liberally thresholded at P < 0.5), averaged across all trials for a given odor. The pseudocolor scale spans the full range of BOLD percent signal change in each map, from minimum (*blue*) to maximum (*red*). Each odorant elicited a distributed pattern of fMRI activity in PPC (*white outline*) that overlapped with, but was distinct from, the other odorants. (*c*) Discriminability of odor-evoked response patterns in PPC. (*Top*) Odor identification accuracy (mean ± between-subjects SEM; *n* = 6) calculated using the mean fMRI activity levels. Odor types could not be differentiated from one another using mean fMRI activity in any of the measured regions of interest. (*Bottom*) Odor identification accuracy calculated using fMRI patterns of ensemble activity exceeded chance level in PPC and OFC. Abbreviations: A, anterior; Amyg, amygdala; APC, anterior piriform cortex; BOLD, blood-oxygen-level-dependent; fMRI, functional magnetic resonance imaging; L, lateral; M; medial; OFC, orbitofrontal cortex; P, posterior; PPC, posterior piriform cortex; SEM, standard error of mean. Figure adapted with permission from Howard et al. (2009).

Early studies were predominantly guided by univariate analysis methods, whereby the fMRI signal was averaged across voxels, scans, and subjects. Pattern-based fMRI analysis methods (i.e., multivariate or multi-voxel pattern analysis), on the other hand, have offered a finer-grained analysis of spatial patterns of BOLD activity (Haxby et al. 2001). Using this novel approach we investigated whether human posterior piriform cortex (PPC) supported spatial ensemble coding of odor qualities and categories (Figure 4). During scanning, subjects sniffed four easily distinguishable odorants. We found that odor classification could be reliably attained from multi-voxel fMRI patterns in PPC and OFC, but not from mean fMRI responses. Furthermore, our findings showed that multi-voxel response patterns were more similar for odorants belonging to the same perceptual categories (e.g., minty odors) compared to odorants in perceptually distinct categories (e.g., woody odors), highlighting that human piriform cortex can represent odor information as distributed activity patterns, consistent with what has been shown in rodents (Stettler & Axel 2009). Together, these results suggest that perceptual and categorical similarity may be a key organizing feature of odor codes in PPC, akin to how visual objects are organized in inferotemporal cortex.

An influential notion in sensory processing posits that expectations of forthcoming stimuli can facilitate encoding of sensory information and can improve the speed and accuracy of perceptual judgments (Friston 2018, Huang & Rao 2011, Summerfield et al. 2006). Freeman and colleagues proposed that odor predictions give rise to odor templates in the brain that mediate odor responses upon encountering the stimulus (e.g., Freeman 1979). To probe whether the human olfactory system implements such mechanisms, we devised a simple template matching paradigm (Zelano et al. 2011). On each trial, subjects were given the name of an odor (i.e., rose or mint) which either matched or mismatched the subsequent odor presentation. The subjects were asked to report whether the odor label and the probe odor were matched at the end of each trial. Notably, we saw that odor-specific templates emerged in piriform cortex prior to the onset of the odorant, and these predictive representations in PPC gave way to post-stimulus representations of the odor itself. Furthermore, the fact that target-related patterns in PPC predicted behavioral performance suggests that the human olfactory system generates predictive templates or “search images” in PPC that may facilitate subsequent odor perception.

While odorants can trigger innate approach or avoidance behaviors, predominantly in nonhuman species (Stowers & Logan 2010), the majority of odors acquire their meaning through learned odor–outcome associations, such as the scent of a food item that comes to be associated with the food source and its complex taste and texture profile. How such associations are acquired and how this process may alter sensory representations have been areas of great interest. Inspired by this question, we conducted an aversive conditioning paradigm relying on pairs of odor enantiomers (mirror-image molecules) that were perceptually indistinguishable at baseline (Li et al. 2008). After pairing one of the two enantiomers with a mild foot shock, the subjects were able to discriminate the conditioned and the unconditioned enantiomer from one another. Paralleling the perceptual divergence between the two mirror-image molecules, we observed that the sensory representations of the two enantiomers, as assessed by piriform multi-voxel ensemble patterns, became more distinct from one another. These findings indicate that learning of novel odor–outcome associations may alter odor representations in piriform cortex, which in turn may facilitate finer discrimination of olfactory cues to shape sensory-guided avoidance behaviors.

Perhaps the most significant perturbation to the olfactory system is a partial or complete loss of smell, as occurs not uncommonly in the context of allergic, viral, and chronic sinus conditions. Using a sensory deprivation paradigm whereby subjects were affixed with nose plugs for 7 days, we investigated how the loss of olfactory input modulates the olfactory system in healthy subjects (Wu et al. 2012). We obtained behavioral and imaging data at baseline, post-deprivation, and at recovery (one week after the end of deprivation). Intriguingly, the deprivation period had no major impact on odor detection and discrimination behavior, but there were reversible changes in both piriform and orbitofrontal odor codes. In particular, multi-voxel ensemble codes of odor quality in OFC became decorrelated after odor deprivation, suggesting that such transient changes in higher-order olfactory regions may support the integrity of odor percepts in the wake of disrupted sensory input. A recent study by Iravani et al. (2021c) showed that subjects who had acquired anosmia (loss of smell) within the past three years demonstrated differences in both gray matter volume and functional connectivity in olfactory as well as nonolfactory regions compared to the aged-matched control group, suggesting that absence of smell can induce plasticity in regions extending beyond the olfactory regions.

### Odor Valence and Odor-Guided Behavior

Classical conditioning paradigms have been a powerful conceptual and empirical tool for olfactory and nonolfactory questions alike. In the olfactory realm, such studies have addressed fundamental questions about how newly formed associations affect odor coding and perception, as discussed above. Notably, however, what is missing from these stimulus-response paradigms (S-R), to put it bluntly, is choice, as classical conditioning does not require an action from the subject. More complex models in the form of stimulus-response-outcome (S-R-O) learning, also referred to as instrumental or operant conditioning, have been lacking in human olfaction studies. This framework involves active engagement in the task, with outcomes based on how, when, and why the subject chooses to act (Balleine & Ostlund 2007), and it offers a more comprehensive assessment of goal-directed behaviors in comparison to classical conditioning or habit formation.

In recent work, Howard & Kahnt (2017) utilized appetizing food odors to assess how OFC represents expected rewards and how these reward representations change when one of the appetizing food odors is devalued through sensory-specific satiety. In this paradigm, the subjects had the choice of receiving either the high- or low-intensity version of one of two odors before and after being satiated with a meal. When subjects were in a state of hunger, both odors were rated to be highly pleasant, and the subjects preferred to smell the high-intensity version of both odorants. After being sated with a food item corresponding to one of the odors (e.g., potato chips), participants instead preferred the low-intensity version of that food odor (e.g., potato chip odor). Consistent with the changes in the reward value of the sated odorant, and paralleled by the subject’s behavioral choice, the representations for the sated odor, but not the unsated odor, were altered in OFC (specifically, lateral posterior OFC) following this selective devaluation. Additionally, the authors observed that the functional connectivity between OFC and ventromedial prefrontal cortex (vmPFC) reflected expected reward and held relevance for the subjects’ choice. These findings illustrate that OFC encodes subjective odor value and can dynamically alter these value representations as a function of internal state while reflecting goal-directed behavior at the individual subject level.

Of course, goal-directed behaviors in the olfactory realm extend beyond the pursuit of odor rewards and can encompass a wide array of targeted behaviors, such as maternal bonding, hunting, feeding, kinship, and avoidance of threats. These behaviors, in animals and humans alike, can utilize odor cues in order to navigate toward or away from the odor source of interest. Studies in rodents have made groundbreaking discoveries of place cells, grid cells, and head direction cells (among others), unveiling neural mechanisms of spatial navigation (Moser et al. 2008). In particular, the discovery of grid cells (Hafting et al. 2005), whose receptive fields tile the environment in a sixfold (hexagonal) symmetric manner in the medial EC, provides a mechanism for cognitive maps of physical space (Behrens et al. 2018). The basic idea is that when moving through a physical space, the movement trajectories that are optimally aligned with the grid orientation will trigger more robust responses than other movement trajectories that are misaligned to the grid orientation. These compelling findings in rodents took on new interest in 2010 when Doeller, Barry, and Burgess combined an fMRI virtual reality paradigm with novel computational methods and identified grid-like representations in the human entorhinal cortex, posterior and medial parietal cortex, lateral temporal cortex, and medial prefrontal areas (Doeller et al. 2010). This pivotal experiment set the stage for investigating the neural mechanisms of spatial navigation in the human brain using noninvasive approaches.

**Figure 5:**
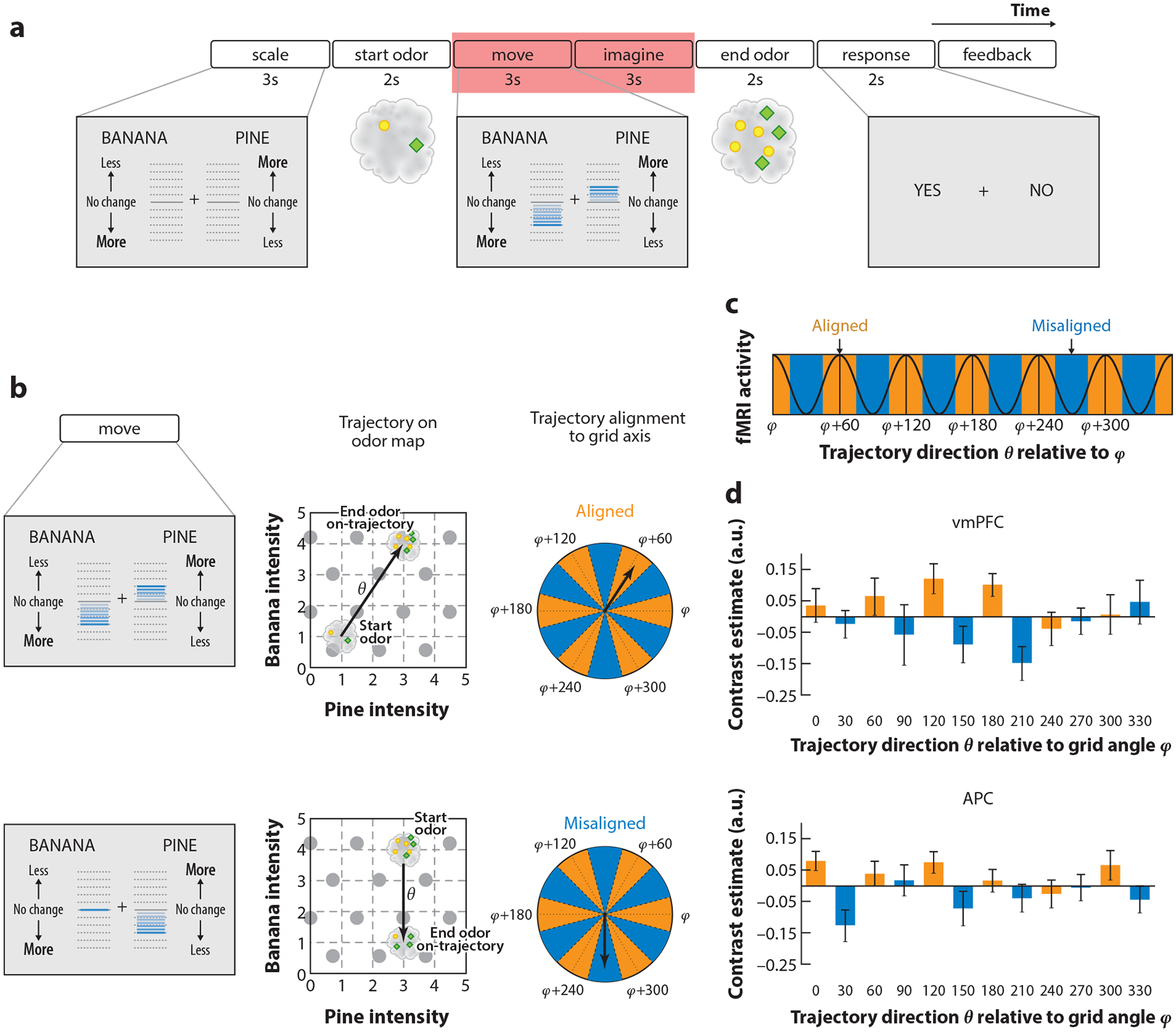
(*a*) Timeline of an example trial of the odor prediction task. The red box indicates the time period used for the grid-cell analyses; the relative movements of the banana and pine scale bars (compare *left* and *center gray boxes*) informed subjects of how much to expect the intensities of the two odor components to change; the subjects then indicated whether the end odor matched their prediction (*right gray box*). (*b*) The experimental design yielded four unique behavioral outcomes, depending on trajectory alignment (aligned or misaligned) and response type (correct versus incorrect). Here we illustrate the two trial types. (*Top row*) Trial in which movement direction *θ* is aligned with hypothetical grid axis angle *φ* and the end odor lies on the trajectory. (*Bottom row*) Same as the top row, except the end odor lies off the trajectory and the movement direction *θ* is misaligned to the hypothetical grid axis angle *φ*. The small gray circles on the 6 × 6 grid represent a putative grid cell field with sixfold symmetry. (*c*) Given a hexagonal grid field with main axis angle *φ*, trajectories on the odor map can be binned as aligned or misaligned with *φ*. Grid-like fMRI activity with 60° periodicity would thus be higher for aligned compared to misaligned trajectories (angle *φ* modulo 60° versus angle *φ* +30 modulo 60°). (*d*, *top*) Cross-validation analysis of the grid-like effect in ventromedial prefrontal cortex (vmPFC) reveals grid angle reproducibility across time (aligned > misaligned). The orange and blue bars indicate alignment and misalignment to *φ*. (*Bottom*) The preferred grid angle in vmPFC predicted hexagonally modulated signal in anterior piriform cortex (APC). Figure adapted with permission from Bao et al. (2019).

Inspired by this new line of work (Bellmund et al. 2018, Doeller et al. 2010), we asked if navigating an abstract cognitive olfactory space could recruit grid-like representations in the human brain (Figure 5). We implemented this abstract olfactory space using two independent axes: One axis varied by the perceived intensity of a banana smell (from zero to high intensity) and the other axis varied by the perceived intensity of pine smell (zero to high intensity) at six different intensity steps in both *x* and *y* directions (Bao et al. 2019). On each trial, participants were presented with an initial odor mixture (composed of pine and banana) and instructed to mentally navigate toward a different target odor mixture (composed of different proportions of pine and banana). At the fMRI level, we identified grid-like representations in the vmPFC, entorhinal cortex, and anterior piriform cortex when subjects were mentally navigating between the initial and the target odor mixtures.

Recent work in our lab by Raithel et al. (2023) investigated whether subjects navigating olfactory landmarks in a virtual reality (VR) setting would display grid-like representations. Here, eight distinct odors were positioned within a VR arena, and the relative position of the odors with respect to one another remained constant throughout the experimental session. On each trial, the subjects were asked to navigate from an initial odor to a target odor, which required the subjects to build an olfactory cognitive map of the arena in order to solve this task efficiently. We have found that navigation of olfactory landmarks elicits grid-like representations in the entorhinal cortex and anterior piriform cortex. Interestingly, grid-like representations in these two regions were aligned to the same grid orientation, suggesting that these two regions may work together to support odor-guided navigation.

Collectively, these findings suggest the presence of olfactory grid-like codes in the human brain and underscore a potential mechanism by which odor information can be compiled into cognitive maps in service of orienting a human navigator toward an odor source.

### Odor-Cued Learning During Sleep

One of the exciting areas of research emerging in the last 15 years has taken place at the intersection of olfaction, learning and memory, and sleep (Arzi et al. 2012, 2014; Bar et al. 2020; Bhutani et al. 2019; Hauner et al. 2013; Knötzele et al. 2023; Perl et al. 2016; Rasch et al. 2007; Rihm et al. 2014). Specifically, targeted memory reactivation (TMR) studies have been central to this new line of research. Simply put, TMR studies deliver a sensory cue during slow-wave sleep to reactivate the cue-associated memory trace and improve memory consolidation. As odorants can be delivered readily without disturbing sleep (Arzi et al. 2012) and can access cortical, limbic, and memory networks without the obligatory thalamic gating, olfactory stimuli are uniquely suited for probing learning and memory during sleep states.

In a landmark study, Rasch et al. (2007) tested whether covert odor cues delivered during sleep could promote consolidation of odor-associated memories and enhance learning. Here, the authors delivered a rose odor while subjects performed a declarative memory and a procedural memory task. Shortly after the learning paradigm (~1.5–2.5 hours), the subjects went to sleep in the laboratory while the investigators delivered rose odor during either slow-wave sleep or rapid-eye-movement (REM) sleep. Presenting the rose odor during slow-wave sleep selectively improved declarative memory (but not procedural memory) compared to the conditions where rose odor was presented during REM sleep or when the odor delivery was omitted. Further control experiments demonstrated that odor exposure was ineffective at improving memory if the odor was presented during wakefulness or if the odorant was not paired with the learning context. These findings provided strong evidence that contextual odor cues can promote memory reactivation and consolidation during slow-wave sleep.

In related work, Arzi et al. (2012) asked whether humans could learn new information during sleep. The authors took advantage of the observation that humans tend to take larger sniffs in the presence of pleasant odors and smaller sniffs in the presence of unpleasant odors. To this end, the authors performed a partial-reinforcement trace-conditioning paradigm whereby two different auditory tones were paired with either pleasant or unpleasant odors while the subjects were asleep. After the conditioning period, and while still asleep, subjects sniffed more vigorously in response to tones predicting a pleasant (versus unpleasant) odor, suggesting that subjects learned the tone–odor associations. Importantly, learning took place outside of the subjects’ awareness of it, and the learned tone–odor associations persisted in the ensuing wake periods.

Further work by Rihm et al. (2014) examined whether odor stimuli could modulate the neural dynamics during sleep to facilitate memory consolidation. The authors observed that presentation of contextual odor cues during sleep increased the power of delta (1.5−4.5 Hz) and fast spindle activity (13–15 Hz), consistent with subsequent work by Perl et al. (2016). These findings suggest that odors may mediate memory consolidation via enhancement of slow-wave oscillations during sleep.

## TEMPORAL DYNAMICS IN THE OLFACTORY SYSTEM

The journey of an odor molecule beginning at the odor source ends shortly after its encounter with the odor receptors. Yet, the cascade of neural activity initiated at the OSNs can persist long after the odorant has left the nasal cavity and can shape neural dynamics on both short (milliseconds to seconds) and long (minutes to days to years) timescales. How odor responses unfold over time, both within and across brain regions, and how odor responses interact with the ongoing neural dynamics are fundamental questions for understanding how the brain processes odor information. While questions in this realm have attracted much attention in the rodent olfactory system, there have been relatively few studies investigating the temporal dynamics of odor responses in the human olfactory system.

The scarcity of knowledge regarding this question mainly arises from the limited temporal resolution of fMRI, the most commonly employed method for investigating the neural basis of human olfaction. In contrast to fMRI, which measures the hemodynamic response occurring on the order of seconds (Logothetis 2008, Rosen et al. 1998), scalp and intracranial electroencephalogram (iEEG) recordings measure the summation of electrical fields generated by neural activity with millisecond resolution (Cohen 2017, Jackson & Bolger 2014). However, when it comes to the olfactory system, scalp EEG faces technical limits for source localization, as the key olfactory structures, including OB, olfactory cortex, and OFC, are positioned distally to the scalp electrodes (Cohen 2017). Furthermore, the skull and the surrounding tissue act as a low-pass filter, limiting the ability to resolve high-frequency activity with scalp electrodes (Buzsáki et al. 2012). On the other hand, iEEG recordings obtained from human patients with medically refractory epilepsy provide an alternative, albeit invasive, approach to record local field potentials directly from olfactory-related regions of interest. Interestingly, recent studies have developed and implemented novel scalp EEG-based methods to record human OB activity by positioning EEG electrodes above the nasal bridge (Iravani et al. 2020, 2021a,b,d). Such studies might bring much needed insights into the function of the human OB.

### Nasal Respiration Patterns Activity in the Olfactory System

Humans, along with all other terrestrial mammals, explore the olfactory world around them through sniffing; each sniff captures a snapshot of the nearby odor molecules, bringing them into the depths of the nasal cavity where the ORs reside (Kepecs et al. 2006, Mainland & Sobel 2006). As such, access of odor molecules to the ORs is shaped by the timing, speed, and frequency of nasal inspirations (Kepecs et al. 2006, Mainland & Sobel 2006, Verhagen et al. 2007, Wachowiak 2011). Due to this inextricable link between orthonasal olfaction and nasal breathing patterns, an overview on the temporal dynamics in the olfactory system reasonably begins with how breathing formats nasal air flow, odor inputs, and consequently the neural responses in the olfactory pathway.

In the 1940s, Adrian (1942, 1950) showed that nasal respiration entrains neural oscillations in the olfactory pathway of hedgehogs. Subsequent studies reported respiration-entrained neural oscillations in the OSNs, OB, and piriform cortex (Carey et al. 2009, Fontanini & Bower 2005, Fontanini et al. 2003, Freeman 1959, Verhagen et al. 2007, Wilson 1998) and, perhaps more surprisingly, have shown that nasal respiration can entrain neural oscillations in regions beyond the ascending olfactory pathway, including the hippocampus (Chi et al. 2016, Lockmann et al. 2016, Yanovsky et al. 2014), barrel cortex (Ito et al. 2014), and prefrontal cortex (Biskamp et al. 2017, Moberly et al. 2018) in rodents.

**Figure 6:**
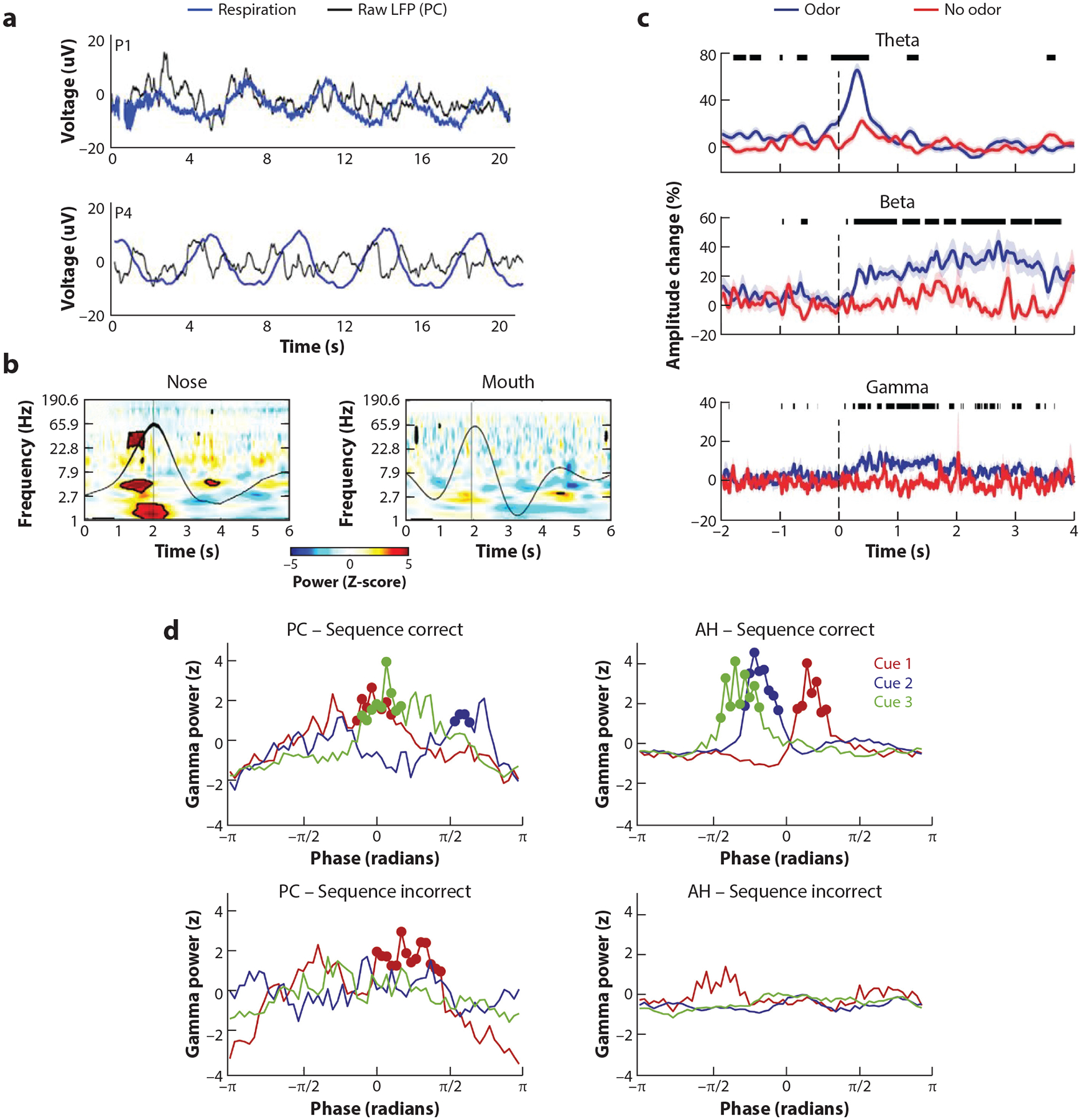
(*a*) Slow oscillations in human piriform cortex (PC) are in phase with respiration. Representative traces of the raw local field potentials (LFPs) from two patients with PC coverage show that slow fluctuations in PC (*black lines*) are in phase with inhalation (*blue lines*) across a series of breaths. Inspiration is in the upward direction. Panel adapted from Zelano et al. (2016). (*b*) Dependence of respiration-induced oscillations on nasal airflow. Time-frequency plot in one representative subject illustrates that respiratory-induced oscillations in PC diminish when breathing is diverted from the nose to the mouth. Panel adapted from Zelano et al. (2016). (*c*) Time course of PC odor-induced oscillations in an odor identification task. Odor-induced theta, beta, and gamma oscillations have different time courses relative to sniff onset. Percent amplitude change from baseline is shown over time series for odor (*blue lines*) and no-odor (*red lines*) conditions. Time = 0 indicates sniff onset, and the thick black lines above indicate statistically significant differences between the odor and no-odor conditions. Panel adapted from Yang et al. (2022) (CC BY 4.0). (*d*) Odor position within a sequence of odors is encoded with theta-gamma code. The position of the odorant within a sequence, independent of its identity, was reflected in the phase of the odor-evoked gamma oscillations with respect to the ongoing theta oscillations in both anterior hippocampus (AH) and piriform cortex (PC). This theta-gamma code was only present in trials in which subjects correctly remembered the position of the odor within the sequence (*top row*) and was not observed in the incorrect trials (*bottom row*). Panel adapted with permission from Yang et al. (2021).

The extent to which breathing influences neural dynamics in the olfactory and nonolfactory regions in the human brain had been underexplored until the last few years. Zelano et al. (2016) recorded iEEG signals from human piriform cortex, amygdala, and hippocampus in patients undergoing epilepsy monitoring to assess how nasal respiration shapes neural oscillations. Findings revealed that neural oscillations in all three regions were modulated by nasal respiration, albeit in different ways. Slow oscillations in piriform cortex were entrained to the respiratory rhythm that occurred in the 0.15–0.35 Hz range (Figure 6*a*). Additionally, the authors observed that nasal respiration modulated the amplitude of higher frequency oscillations, with significant increases in the power of delta, theta, and beta oscillations, all aligned to the onset of inhalation. Notably, when the subjects were instructed to switch from nasal breathing to mouth breathing, the entrainment effects were greatly diminished, consistent with previous rodent studies (Figure 6*b*).

Subsequent iEEG studies by Herrero and colleagues have expanded on these findings, revealing coherence between nasal respiration and neural oscillations in OB, piriform cortex, OFC, amygdala, and hippocampus as well as many other cortical and subcortical regions, suggesting that nasal respiration may entrain neuronal oscillations across a wider range of brain regions than previously shown, including structures outside of the olfactory pathway (Herrero et al. 2018). Similar brain-wide effects of nasal respiration were also seen in noninvasive magnetoencephalography recordings from healthy subjects (Kluger & Gross 2021). Additional behavioral studies have shown that the phase of nasal respiration modulates task performance in both olfactory (Arshamian et al. 2018) and nonolfactory (Kluger & Gross 2020, Kluger et al. 2021, Nakamura et al. 2018, Perl et al. 2019, Zelano et al. 2016) paradigms, suggesting that respiration-entrained fluctuations in neural excitability can modulate task-relevant neural processes.

Together, these findings suggest that ongoing neural activity in olfactory and nonolfactory brain regions can be structured by nasal respiration in the human brain. Although the function and behavioral relevance of this entrainment are areas of active study, it has been hypothesized that these widespread rhythms arising from nasal breathing can structure the excitability of neural populations and coordinate neural activity across brain regions in a task-dependent manner (Heck et al. 2017, Tort et al. 2018).

### Temporal Dynamics of Odor Responses

Early intracranial experiments investigating odor responses in the human brain began more than five decades ago. These studies were primarily focused on the amygdala (Hudry et al. 2001, 2003, Hughes & Andy 1979, Jung et al. 2006), a region with extensive connectivity to olfactory structures (Carmichael et al. 1994, Noto et al. 2021, Zhou et al. 2019a), and sampled commonly during intracranial monitoring of epilepsy patients (but see Hughes et al. 1969, 1970 for OB recordings). The authors observed that odors elicited oscillatory responses tiling the inhalation period and reasoned that these oscillations reflected olfactory processing, as the responses were odorant specific (Hughes & Andy 1979) and sensitive to repetition suppression (Hudry et al. 2003, Jung et al. 2006).

Intracranial characterization of odor responses in piriform cortex only emerged in the past six years, nearly four decades after odor-evoked oscillations were observed in the amygdala. Jiang et al. (2017) conducted an odor detection task while monitoring piriform local field potentials with intracranial depth electrodes. On each trial, the subjects received one of four distinct odors or odorless air and reported if an odor was present. Odors, in contrast to odorless air, elicited robust theta oscillations across all subjects and odor types. The features of these theta oscillations (e.g., peak frequency, timing, and power) were distinguishable across odors, and a classifier trained on theta oscillations could successfully decode odor identity, which emerged as early as ~110 milliseconds after inhalation onset. These findings suggest that human piriform cortex can rapidly differentiate across odorants using information in the time-frequency domain.

Experiments utilizing more complex olfactory paradigms have also revealed beta, gamma, and theta oscillations (Dikeçligil et al. 2023; Yang et al. 2021, 2022). Notably, the time course of these odor-induced oscillations was consistent across studies, such that theta typically spanned the first ~500 millisecond interval after odor onset, with beta and gamma oscillations emerging at the tail end of theta oscillations and persisting for as long as ~3 seconds (Figure 6*c*). A potential explanation for different oscillatory dynamics across studies might be the differences in task demands. It is possible that theta oscillations may reflect an initial stage of odor processing in piriform cortex, as they emerge early across both simple and complex olfactory paradigms. On the other hand, the faster oscillations (i.e., beta and gamma) observed during more cognitively demanding paradigms may reflect higher-order processing, given that they emerge later and can persist for multiple seconds. Yang et al.(2022) investigated whether oscillations in piriform cortex are also associated with task performance in olfactory paradigms. The authors found that the presence of gamma, but not theta, oscillations during odor sampling was predictive of successful odor identification, consistent with the idea that fast oscillations may support higher-order olfactory processing in humans.

A seemingly ubiquitous neural dynamic observed across many brain regions, tasks, and species is the modulation of the faster oscillations (i.e., gamma) by the phase of slower oscillations (e.g., theta) (Lisman & Jensen 2013). One hypothesized function of the coupling of theta and gamma rhythms emerged from the memory literature, suggesting that the phase of gamma oscillations within the theta cycle can provide additional information and act as a neural code to organize multiple items in working memory (Lisman & Jensen 2013).

In work from our lab, Yang et al. (2021) investigated whether theta-gamma coupling in piriform could support working memory of olfactory content. In an olfactory version of a Sternberg paradigm, the subjects were presented with three consecutive distinct odors. After a ~10 second waiting period, the subjects were asked to assess if a fourth odor (i.e., probe odor) matched one of the original three odors, and if so, what the position of the odor was within the sequence. We observed that gamma oscillations occurred at distinct phases of the underlying theta cycle depending on the position of the odor within the sequence (i.e., first, second, or third). Interestingly, this theta-gamma encoding of sequence position was only observed when subjects could correctly recall the temporal order of odors.

Olfactory iEEG experiments emerging in recent years present the first clues on how different regions may interact during odor processing. In the simple odor detection paradigm discussed above (Jiang et al. 2017), the authors observed that theta oscillations between piriform cortex and anterior hippocampus were phase locked upon odor onset, suggesting that this phase locking may facilitate information transfer between the two regions. A separate iEEG study investigated the interregional dynamics between auditory and olfactory cortices during an auditory cued odor identification task (Zhou et al. 2019b). The authors observed increased phase synchrony between piriform and auditory cortices after the auditory cue, and the presence of phase synchrony was associated with correct task performance. These results suggest that phase synchrony across olfactory and nonolfactory areas could be an important mechanism for integrating task-related information across regions.

## CONCLUSIONS

The main goal of this review article has been to convey the sheer fecundity of research currently taking place in the area of human olfaction. The scientific foundations of smell began with Aristotle, followed by those semi-alchemist botanists and lute-plucking troubadours of scent emerging over the next couple hundred years, but by the 1930s, the nascent field of modern-day neuroscience was emerging, and at the forefront, olfactory-wise, was Lord Adrian, with his detailed investigations of the hedgehog, an animal uniquely chosen due to the ease of surgical access to piriform cortex (Adrian 1942, 1950). Out of this framework arose a few decades focused on a plethora of animal model systems, biological underpinnings of G-protein coupled receptors, and of course the triumph of identifying the genetic family of ORs. Translation from the burgeoning neuroscience of animal models to the piecemeal neuroscience of human studies was limited mostly to anatomy and behavior, but as this review has shown, the subsequent emergence of new methods and technologies—hand in hand with a growing scientific understanding of how to characterize the neuroscience of smell and even to generate causal inferences via transcranial magnetic stimulation (Howard et al. 2020, Jadauji et al. 2012)—has introduced even greater formalism to the questions that can be asked of human olfaction. While we have specifically focused this review on a handful of conceptual themes, from molecules and receptors to olfactory ensemble coding to the millisecond timescale of local field potential recordings in patients with medically refractory epilepsy, we recognize that with a finite word limit, we were unable to do justice to the many other compelling research studies not discussed here.

There remain important technical and conceptual challenges in the field of human olfactory neuroscience. While most of our current understanding regarding the neural basis of olfaction continues to be derived from rodent studies, there are distinct challenges in working with animal models to assess odor percepts and their relationship to underlying neural activity. Given that animals cannot talk, odor percepts are instead inferred based on animals’ behavioral responses on tasks such as go/no-go and two-alternative forced-choice paradigms. Additionally, these paradigms typically require extensive training, often involving reward- and punishment-based incentives that may introduce an additional layer of potential confounds that need to be taken into account when analyzing olfactory responses. This is where human studies provide important advantages for studying odor perception, as humans can readily report their odor experience and take part in complex behavioral paradigms with minimal training. However, naming and identification of odor stimuli do not come easy to the untrained nose and can be variable, both within and across subjects. Compared to animal models, human studies are confronted with a different set of methodological challenges, as experiments are constrained by the technical limitations of noninvasive approaches. Notably, the burgeoning frontier of intracranial recordings has opened an exciting avenue for characterizing the time course of odor responses within and across brain regions at millisecond resolution. While new techniques and analysis methods push the boundaries of noninvasive studies, functional imaging of the human OB and single neuron recordings from human olfactory structures remain elusive.

Despite the significant advances summarized in this review, the majority of the human ORs remain orphaned. Furthermore, the boundaries of the olfactory stimulus space are currently underdefined, and there is no consensus in the field regarding the number of possible odorous molecules the human nose can detect. One novel direction currently emerging in the field is the effort to develop and implement an “electronic nose.” Successful implementation of such an odormachine interface embedded into the olfactory nasal cleft would be a foundational paradigm shift, especially if the interface were able to reproduce stable, specific, and robust odor percepts.

Both the neuroanatomy and the synaptic connectivity of the human olfactory system remain understudied, and the axonal projections between the olfactory, limbic, and cognitive regions remain poorly understood. One approach has been to rely on functional connectivity at resting states to determine correlated activity across regions as a proxy for direct or indirect synaptic pathways. However, the extent to which these resting-state correlations reflect bona fide synaptic connections remains unknown. Postmortem neuroanatomical studies that carefully dissect and assess the axonal pathways would provide much needed insights into the similarities and distinctions between the human and rodent olfactory systems.
